# Research on Adhesion Performance of Silicone Gel for Power Module Packaging Regulated by Crosslink Structure and Interfacial Connection

**DOI:** 10.3390/gels12070647

**Published:** 2026-07-19

**Authors:** Xiangze An, Dongxin He, Xiaobin Zheng, Tinghui Li, Cong Zhang, Hongshun Liu

**Affiliations:** School of Electrical Engineering, Shandong University, Jinan 250061, China; 202000190125@mail.sdu.edu.cn (X.A.); 202534828@mail.sdu.edu.cn (X.Z.); litinghui@mail.sdu.edu.cn (T.L.); lhs@sdu.edu.cn (H.L.)

**Keywords:** high-voltage power module, silicone gel, adhesion performance, crosslink structure, silane coupling agent, breakdown strength

## Abstract

Silicone gel for high-voltage power module packaging is prone to interfacial failure due to poor intrinsic adhesion, which seriously threatens the reliability of devices. This study explores ways to improve the adhesion performance of silicone gel from the two aspects of crosslink network structure and interfacial connection. The crosslink structure is regulated by adjusting the ratio of side-hydrogen-containing silicone oil to terminal-hydrogen-containing silicone oil, and interface adhesion is improved by adding three different contents of silane coupling agents (KH560, KH570, A171). The adhesion strength is evaluated by lap shear experiments. The results show that when the ratio of side-hydrogen to terminal-hydrogen is 16:24, the adhesion strength reaches a peak value of 0.0921 MPa. Among the coupling agents, KH560 shows the most significant enhancement, with the adhesion strength reaching 0.1356 MPa at 4% addition and a 47% improvement over the baseline, KH570 is only effective at low addition levels, and A171 shows the weakest effect due to vinyl interference in the crosslink network. Breakdown tests confirm that all three modification schemes do not seriously damage insulation performance. This study provides a feasible strategy and basis for the adhesion reliability design of silicone gel for power module packaging.

## 1. Introduction

In the context of the clean transformation of energy systems, high-voltage power modules have become a core component to ensure the safe and stable operation of new power systems [1]. As high-voltage power modules continue to evolve towards higher voltage levels and higher power density, their packaging reliability is facing increasingly severe challenges [2]. As the core packaging material [3], the performance of silicone gel directly determines the long-term reliability of power modules. Therefore, improving the comprehensive performance of silicone gel has great engineering value.

As the packaging silicone gel forms interfaces with chips, lead frames and substrates in power modules, its adhesion performance directly affects the long-term reliability of packaging. Due to the lack of surface reactive groups and low surface free energy, the existing silicone gel suffers from poor adhesion and weak interfacial bonding [4]. Long-term operation and heat generation can lead to interface delamination, resulting in local electric field concentration, hindering heat dissipation [5], and may induce partial discharge, seriously threatening the lifespan of the module and system stability [6]. Copper components are key areas for adhesion failure due to their large contact area [7,8] and significant interface stress [9]. Therefore, copper is selected as the representative substrate for adhesion testing in this study. While we acknowledge that silicone gel also contacts other materials, copper serves as a practical and conservative representative for the most demanding metal–polymer interfaces, allowing systematic investigation without confounding variables from diverse surface chemistries. Therefore, it is of great importance to improve the adhesion between silicone gel and copper substrates to ensure the stable operation of high-voltage modules. At present, research on packaging insulation materials mainly focuses on improving their own properties such as electrical strength and thermal stability [10], while there is still a lack of exploration of the adhesion behavior between materials and actual interfaces.

At present, silicone gel is usually prepared by the hydrosilylation reaction between vinyl silicone oil and hydrogen-containing silicone oil [11]. By adjusting the crosslink process, the intrinsic crosslink structure of silica gel can be effectively changed, and then its performance can be adjusted. At present, research on the regulation of the crosslink structure of silicone gel mainly focuses on tensile properties [12], surface properties [13], electrical tree development process [14] and thermal stability [15], while systematic research on the influence of crosslink structure on adhesion properties is still lacking. As to how to improve the adhesion between silicone gel and the interface, researchers mostly use the method of adding small molecular adhesion promoters with epoxy, vinyl and other functional groups [16,17,18]. However, adhesion promoters need to be designed and prepared by themselves, which has the disadvantages of complex process and high cost. Other researchers have improved adhesion by treating the surface with plasma, corona discharge and other technologies [19,20], but there are problems such as complex processing, difficulties in application to the internal structure of the package, and treatment effect decays with time. More fundamentally, such surface treatment approaches require direct processing of the internal surfaces of the module, which risks damaging sensitive components such as chips and bonding wires and is inherently difficult to implement uniformly within complex three-dimensional structures. In contrast, incorporating silane coupling agents as integral blend additives is a material-level modification strategy that requires no post-processing of the module, while offering superior process compatibility and manufacturability for practical encapsulation applications. Silane coupling agents have been successfully applied to improve adhesion in various polymer–inorganic interfaces, such as polymer–wood composites [21], thermoplastic polyurethane–steel [22], polyurethane coatings on tin [23] and resin–titanium systems [24]. It is therefore anticipated that this approach can be extended to enhance the interfacial adhesion between silicone gel and module substrates in power module packaging.

Therefore, this paper systematically explores effective ways to improve the adhesion performance of silicone gel from the two aspects of crosslink structure regulation and interface properties. On the one hand, by adjusting the ratio of side-hydrogen-containing silicone oil to terminal-hydrogen-containing silicone oil, the crosslink network topology of the hydrosilylation reaction is changed, and then the effect of the crosslink structure on adhesion strength and failure mode is studied. On the other hand, three kinds of silane coupling agents with different types and contents are introduced to evaluate their effects on improving the adhesion between silicone gel and the metal interface. In this study, the effects of two modification paths of intrinsic crosslink structure optimization and interfacial connection are explored, and the optimal process parameters of each strategy are determined, aiming to provide a variety of options and a theoretical basis for the adhesion reliability design of high-voltage power module packaging silicone gel.

## 2. Results and Discussion

### 2.1. The Influence of Different Crosslink Structures on Adhesion Performance

**Figure 1:**
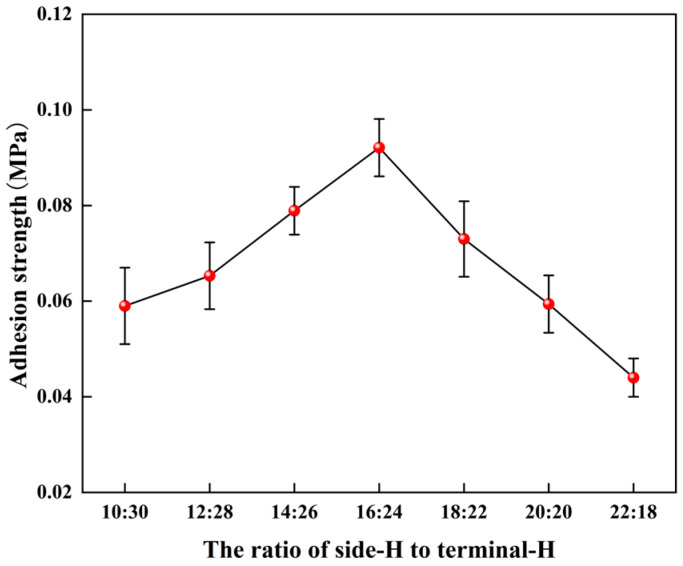
Adhesion strength with different crosslink ratios.

**Figure 2:**
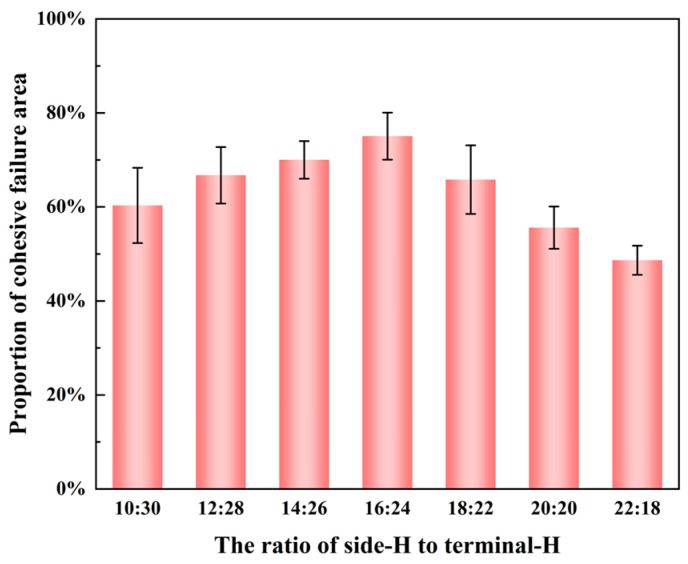
Proportion of cohesive failure area with different crosslink ratios.

Figure 1 and Figure 2, respectively, show the test results of the adhesion strength of silicone gel and the proportion of cohesive failure area with different crosslink ratios. As shown in the figures, as the ratio of side-hydrogen to terminal-hydrogen changes from 10:30 to 22:18, both the adhesion strength and the proportion of cohesive failure area show a trend of first increasing and then decreasing. The peak values appear at a ratio of 16:24, and then both continue to decline. At a ratio of 16:24, the adhesion strength reaches 0.0921 MPa, representing increases of 56% and 109% compared to 10:30 (0.0590 MPa) and 22:18 (0.0440 MPa), respectively. At the same ratio of 16:24, the proportion of cohesive failure area is 75.05%, which is 24% and 54% higher than those at 10:30 (60.32%) and 22:18 (48.65%), respectively. The improvement in adhesion performance is significant when the ratio is 16:24. In addition, it can be seen from the graph that the change in adhesion strength and the proportion of cohesive failure area show a similar trend.

**Figure 3:**
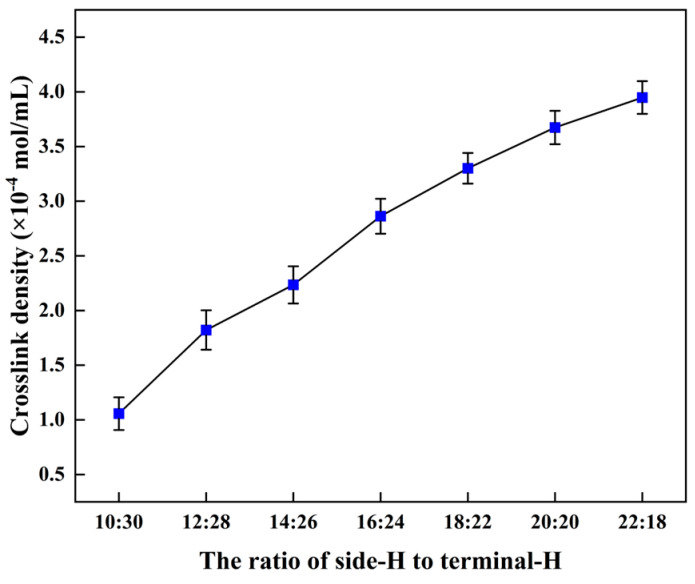
Crosslink density with different crosslink ratios.

This trend is due to the synergistic regulation of interface adhesion and intrinsic mechanical properties by the crosslink network structure. The crosslink density with different crosslink ratios is shown in Figure 3. Terminal-hydrogen-containing silicone oil only has Si-H groups at both ends of the molecular chain, and after participating in addition reactions, it mainly forms linear or suspended chain segments, with a low degree of overall crosslink and a large number of free chain ends. When the terminal-hydrogen content ratio is high, the crosslink density of the system is low, which is conducive to full physical contact with the surface of the copper substrate, so as to obtain a certain initial adhesion. However, due to the low proportion of hydrogen in the side, sparse crosslink nodes in the body, and insufficient cohesive strength, cracks are prone to propagate along the interface under external forces. Therefore, the adhesion strength is low when the ratio is 10:30 and 12:28, and the proportion of cohesive failure area is relatively low.

With increase in side-hydrogen content to 16:24, more crosslink nodes are introduced into the side-chain Si-H, and the linear segments in the network gradually change into a three-dimensional structure with moderate crosslinks. The moderate crosslink density can effectively transfer the shear stress to the whole network, avoiding stress concentration in the interface area. Therefore, the interface adhesion strength is improved, and the crack preferentially occurs and expands in the body during failure, so the cohesive failure area reaches the maximum value (75.05%), and the adhesion strength also rises to the peak value (0.0921 MPa).

When the hydrogen content ratio exceeds 16:24, the crosslink density further increases, and the movement of the chain segment is restricted. The high crosslink density leads to an increase in the brittleness of the silicone gel, and the internal stress is difficult to release through the molecular chain. These stresses are concentrated on the interface between the silicone gel and the copper substrate, which readily induces interface failure during shearing. Therefore, when the ratio exceeds 18:22, the adhesion strength continues to decrease, and the cohesive failure area also decreases, indicating that the failure mode has shifted back to interface delamination. The mechanical properties of silicone gel with different crosslink ratios will be discussed in detail in the next section.

In conclusion, the non-monotonic regulation of the adhesion of silicone gel is achieved by adjusting the crosslink structure. The optimal ratio in this system is 16:24, at which the crosslink density and interfacial connection reach a reasonable balance, the adhesion strength is the highest, and the failure mode is mainly dominated by cohesive failure.

### 2.2. The Influence of Different Crosslink Structures on Hardness and Shear Modulus

**Figure 4:**
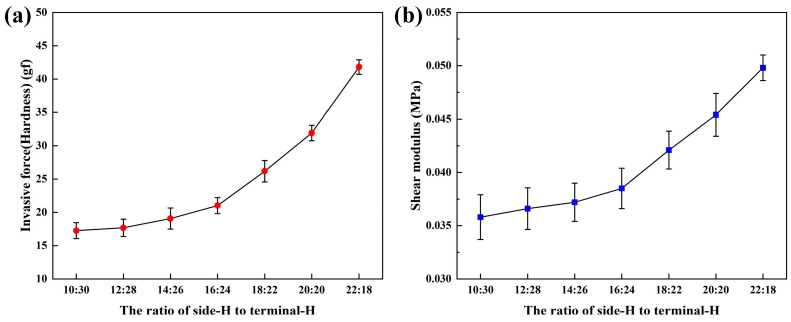
(**a**) Hardness (invasive force) with different crosslink ratios; (**b**) Shear modulus with different crosslink ratios.

The hardness and shear modulus of silicone gels with different crosslink ratios are measured and the results are shown in Figure 4. Both hardness and the shear modulus exhibit a monotonic increasing trend with increase in the side-H to terminal-H ratio. Notably, the increase is relatively gradual for ratios below 16:24, whereas a pronounced rise is observed from 16:24 onward. When the side-H content is low, the network is a relatively loose structure with limited crosslink junctions. Consequently, both hardness and shear modulus increase only modestly with increasing side-H content. However, as the side-H ratio exceeds 16:24, the three-dimensional structure becomes increasingly dense and the shear modulus significantly increases, which is an important manifestation of the significant increase in bulk cohesive strength.

The adhesion strength and cohesive failure proportion reach their maxima precisely at the 16:24 ratio—the very point where the rapid increase in hardness and shear modulus begins. At ratios below 16:24, the relatively low cohesive strength limits the overall adhesion capacity, and failure tends to occur cohesively but at lower stress levels. At 16:24, the crosslink density achieves an optimal balance. The network is sufficiently dense to enable efficient shear stress transfer from the interface into the bulk to dissipate stress. Beyond 16:24, however, the substantial increase in shear modulus and hardness indicates that the network has become overly rigid. The dense crosslink structure significantly prevents stress relaxation and causes internal stress to concentrate at the interface. This stress concentration promotes interfacial failure, manifested as a decrease in the cohesive failure proportion and a corresponding decline in the adhesion strength. Thus, moderate crosslinking enhances adhesion by improving stress transfer, while excessive crosslinking leads to brittleness and interfacial stress concentration that ultimately degrades adhesion performance.

### 2.3. The Influence of Changing Crosslink Structure on Breakdown Performance of Silicone Gel

**Figure 5:**
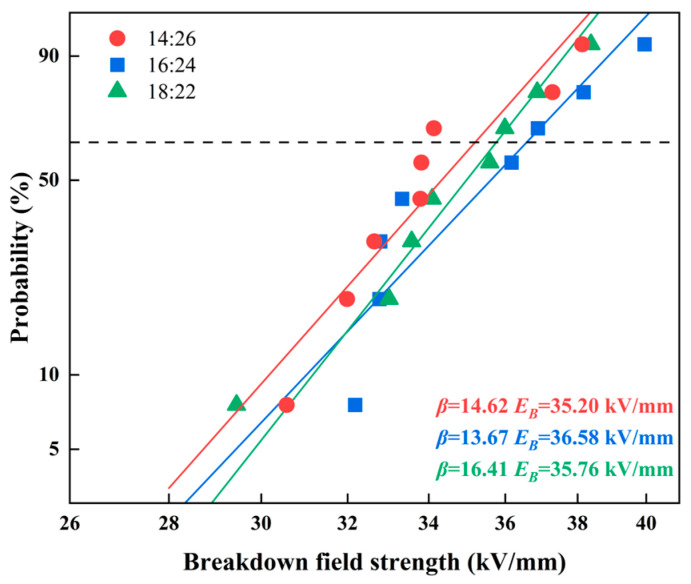
Weibull distribution diagram of breakdown field strength with different crosslink ratios.

Three ratios of 14:26, 16:24 and 18:22 with better adhesion are selected to observe whether changing the crosslink structure would damage the breakdown field strength of silicone gel. Due to the discreteness of the breakdown strength, this study uses the Weibull distribution commonly used in reliability analysis to analyze the experimental data, as shown in Figure 5. The straight line in the figure is a fitting line of the Weibull distribution. Taking the eigenvalues at 63.2% of the Weibull distribution, the breakdown field strengths obtained are 35.20 kV/mm, 36.58 kV/mm and 35.76 kV/mm, respectively. From the numerical values, it can be seen that the breakdown field strength remains relatively high, with a difference of no more than 1.4 kV/mm, and the breakdown field strength does not deteriorate due to changes in the crosslink structure.

### 2.4. The Influence of Different Silane Coupling Agents on Adhesion Performance

**Figure 6:**
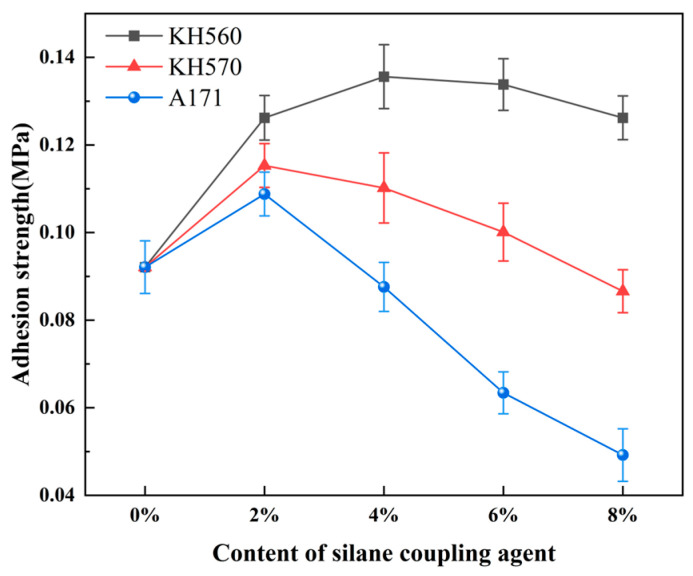
Adhesion strength with different types and ratios of silane coupling agents.

**Figure 7:**
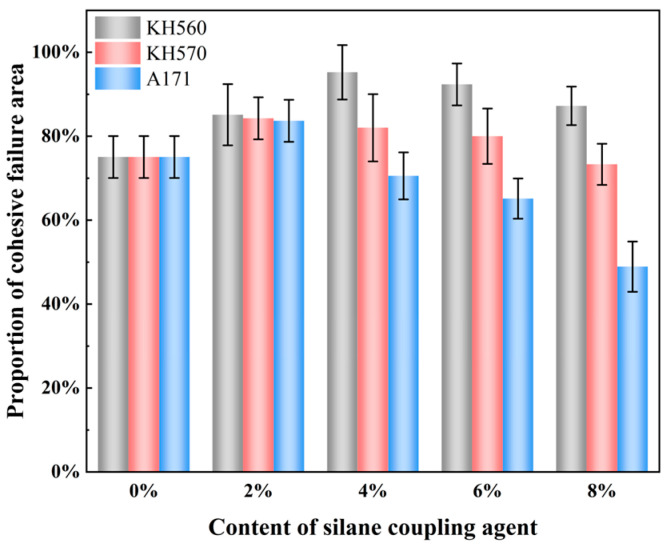
Proportion of cohesive failure area with different types and ratios of silane coupling agents.

**Figure 8:**
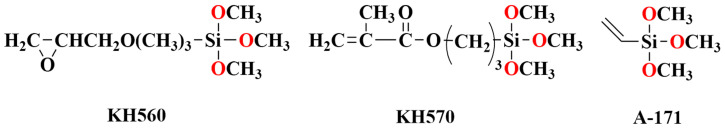
Molecular formulas of three silane coupling agents.

Figure 6 and Figure 7, respectively, show the test results of the adhesion strength of silicone gel and the proportion of cohesive failure area under three different silane coupling agents with different addition ratios. All samples are prepared with a hydrogen content ratio of 16:24 based on the optimal adhesion performance tested earlier. The molecular formulas of the three silane coupling agents are shown in Figure 8.

**Figure 9:**
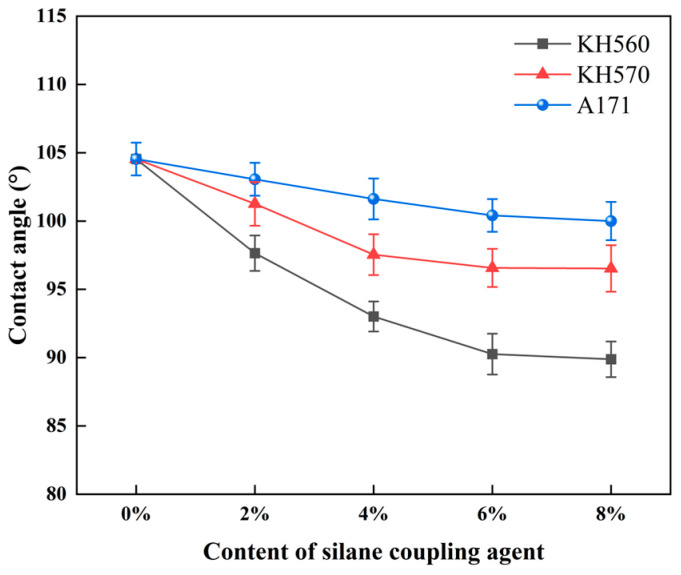
Contact angle with different types and ratios of silane coupling agents.

**Figure 10:**
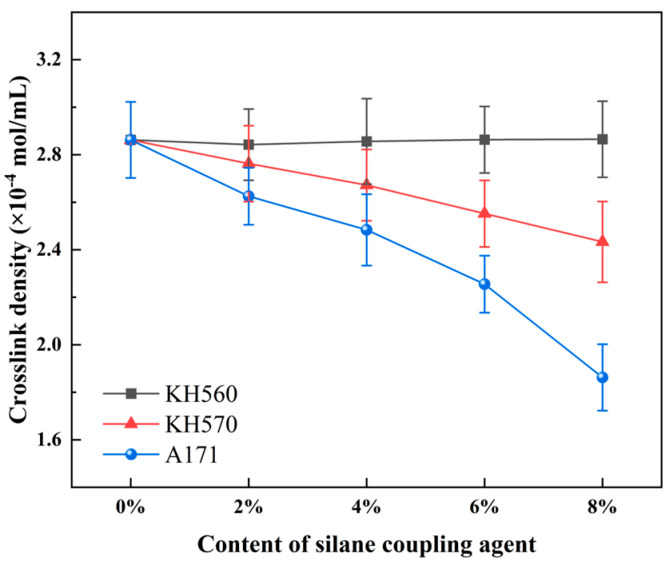
Crosslink density with different types and ratios of silane coupling agents.

For KH560, at all addition levels ranging from 2% to 8%, there is a significant improvement in adhesion strength compared to 0%, with an increase of over 37%, and the proportion of cohesive failure increased to over 85%. At the same time, the contact angle shows a significant decreasing trend with increase in the addition amount, as shown in Figure 9. The figure shows that the introduction of KH560 effectively improves the polarity of the silicone gel surface and improves its wettability to copper plate. According to surface infiltration theory, a lower contact angle means higher surface energy [25,26], and silicone gel can be more fully spread on the copper plate surface, thus improving the strength of physical adsorption and mechanical interlock. On the other hand, the crosslink density test results show that there is almost no change in the crosslink density compared to the 0% sample after adding KH560, as shown in Figure 10. This shows that KH560 does not interfere with the crosslink network of silicone gel, so the cohesive strength of silicone gel can be kept stable. Overall, within the range of 2% to 4% addition of KH560, a significant decrease in contact angle leads to a significant improvement in wettability, while maintaining stable crosslink density. As a result, the adhesion strength continues to increase and reaches its peak at 4%. When the addition level is further increased to 6% and 8%, the adhesion strength shows a slight decrease, yet remains significantly higher than that at 2% addition, and far superior to all KH570 and A171 groups. Therefore, KH560 can effectively enhance adhesion within a large range of addition amounts of 2% to 8%, with the optimal addition ratio being 4% to 6%.

The performance of KH570 exhibits different phenomena. When the addition amount is 2%, both the adhesion strength and the proportion of cohesive failure are improved. At 4%, both indicators slightly decrease. As the amount of addition continues to increase, the adhesion strength and the proportion of cohesive failure continue to decrease, even lower than the control group. Compared with KH560, KH570 has a weak ability to reduce the contact angle, which indicates that KH570 results in limited improvement on the surface wettability of silicone gel. The crosslink density results show that the addition of KH570 leads to a decrease in crosslink density. This indicates that KH570 can interfere with the formation of the originally regular crosslink network. The decrease in crosslink density directly leads to decrease in the cohesive strength of silicone gel. Even if the wettability at the interface is improved to a certain extent, the overall adhesion strength will be limited due to insufficient bulk strength. Specifically, at a low addition level of 2%, the contact angle decreases and the wettability slightly improves. At the same time, the decrease in crosslink density is not yet significant, so the adhesion strength can still be improved to a certain extent. As the amount of addition continues to increase, the crosslink density continues to decrease. Although the contact angle still decreases, the cohesive strength of the body deteriorates and the adhesion strength continues to decrease. Therefore, the effective concentration range of KH570 is relatively small, with a suitable addition amount of 2% to 4%, which can be detrimental to adhesion performance if exceeded.

The results for A171 are the most sensitive. When 2% is added, both the adhesion strength and the proportion of cohesive failure increase. After increasing the addition amount, the adhesion strength and the proportion of cohesive failure decrease significantly. The results of the contact angle show that the decrease is relatively small after adding A171. This shows that A171 improves the wettability of silicone gel very little, and its surface energy enhancement effect is very weak. More critically, the vinyl functional group of A171 can directly participate in the hydrosilylation reaction under platinum catalysis. Since each A171 molecule contains only one vinyl group, it reacts with Si-H in the network to form a hanging chain rather than a chain extension or crosslink node. From a chemical standpoint, this would inevitably disrupt the originally uniform crosslink network and reduce the overall crosslink density. This mechanistic inference is directly corroborated by the crosslink density measurements presented in Figure 10. As the A171 addition level increases from 0% to 2%, 4%, 6% and 8%, the crosslink density exhibits a clear and continuous decreasing trend. It unequivocally confirms that A171 actively participates in the reaction and interferes with network formation. The resulting reduction in crosslink density not only weakens the cohesive strength of the bulk material but also introduces local stress concentration points, further compromising the mechanical integrity of the interface. Although A171 participates in the hydrosilylation reaction via its vinyl group, the resulting small-molecule pendant chains are likely fully buried within the silicone gel bulk rather than enriched at the surface, leaving the surface chemistry largely unchanged, so the change in contact angle is not significant. Overall, A171 can only slightly improve adhesion performance at a low addition level of 2%. Once the addition level exceeds 2%, the negative impact will be severe.

Comparing the mechanisms and effects of three coupling agents, the difference lies in the influence on the surface wettability of silicone gel and on the crosslink network. KH560 does not interfere with the crosslink network and can significantly reduce the contact angle. By improving wettability, it can stably enhance adhesion performance within a wide range of addition amounts. KH570 and A171 both reduce crosslink density and damage the cohesive strength of the body. KH570 results in a certain improvement in wettability, but A171 hardly improves wettability and severely damages the crosslink network, only slightly increasing at 2%. Higher addition levels have serious negative effects.

### 2.5. The Influence of Silane Coupling Agent on Intrinsic Breakdown Performance of Silicone Gel

In the packaging application of high-voltage power modules, silicone gel not only needs to have good interface adhesion performance to ensure mechanical stability and heat dissipation, but also its inherent high-voltage insulation reliability is core to ensuring the long-term safe operation of modules. Although previous research has confirmed that the introduction of a silane coupling agent in proper proportion can improve the interface adhesion of silicone gel to copper plate, any material modification may affect its dielectric strength and other bulk insulation properties. Therefore, it is necessary to evaluate whether a silane coupling agent would damage the breakdown performance of silicone gel.

**Figure 11:**
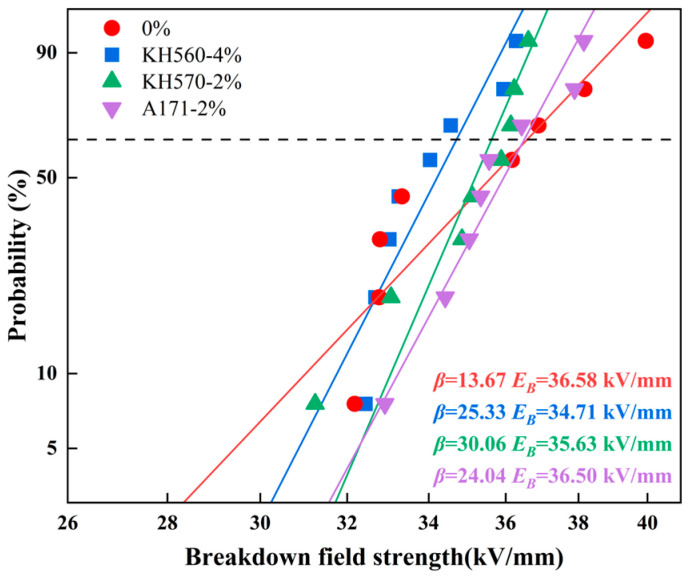
Weibull distribution diagram of breakdown strength with different silane coupling agents.

The breakdown strength tests are conducted on the control sample without added coupling agent and the three groups of samples with the best adhesion performance, KH560-4%, KH570-2% and A171-2%, as shown in Figure 11. The breakdown field strengths of the four samples are 36.58 kV/mm, 34.71 kV/mm, 35.63 kV/mm and 36.50 kV/mm, respectively. It can be seen that the introduction of three coupling agents does not significantly degrade the intrinsic insulation performance of silicone gel.

After adding 4% KH560, the breakdown field strength decreases by only 5.1%. The epoxy group in the KH560 molecule does not react with the hydrosilylation system, but its polarity is stronger than that of the silicone gel main chain. Under the influence of an electric field, it may introduce local polarization centers, leading to local electric field distortion. However, due to the addition of only 4% KH560, its impact on breakdown performance is limited. The breakdown field strength of the sample with 2% KH570 added decreases by about 2.6%. The methacryloyloxy of KH570 may introduce local polarization centers at low addition levels. However, due to the fact that the addition amount is only 2% and the group may partially participate in the reaction or be distributed at the interface, its impact on the overall polarity of the body is limited. The breakdown field strength of the sample with 2% A171 added is almost the same as the control sample. The vinyl group of A171 also participates in hydrosilylation, but its small molecules cause less disturbance to the network uniformity. Moreover, A171 is almost completely consumed in the reaction at low addition levels, further reducing defects. Therefore, the breakdown field strength does not change significantly.

The above results show that the breakdown field strength of the silicone gel is not substantially damaged by the three silane coupling agents within the range of the amount added to optimize the adhesion performance. This verifies that the high voltage insulation reliability of silicone gel can be maintained while the adhesion property is improved by silane coupling agent modification.

## 3. Conclusions

Seeking to address the problem of insufficient adhesion of silicone gel interfaces for high-voltage power module packaging, this paper systematically studies methods of improving the adhesion performance of silicone gel to copper substrate through crosslink structure control and silane coupling agent interfacial connection. The main conclusions are as follows:

(1) The crosslink structure of silicone gel can be effectively changed by adjusting the ratio of side-hydrogen-containing silicone oil to terminal-hydrogen-containing silicone oil. When the hydrogen content ratio is 16:24, the crosslink density and interfacial connection reach an optimal balance, and the adhesion strength and the proportion of cohesive failure area reach an optimal level. Excessive side-hydrogen content leads to a dense crosslink network, concentrated internal stress and decreased adhesion performance. If the proportion is too low, cracks will propagate along the interface due to insufficient crosslinking. The breakdown strength of the silicone gel at three crosslink ratios with excellent adhesion performance still remains high.
(2) Three types of silane coupling agents exhibit different enhancement effects due to their different functional groups. KH560 does not disturb the crosslink network and effectively lowers the contact angle, improving wettability and stably enhancing adhesion over a wide addition range, with an optimum at 4%. KH570 improves adhesion only at 2% low addition, but higher amounts reduce crosslink density and limit wettability, leading to performance deterioration. A171 severely reduces crosslink density while barely lowering the contact angle; only a slight increase occurs at 2%, and adhesion sharply decreases at higher addition levels.
(3) The breakdown field strength of three groups of samples added with a silane coupling agent with the best adhesion performance is tested, and the results show that the introduction of three coupling agents does not significantly degrade the intrinsic insulation performance of silicone gel. This shows that silane coupling agent modification can maintain the high voltage insulation reliability of the silicone gel while optimizing adhesion performance.
(4) Crosslink structure regulation is an intrinsic optimization strategy, and the optimal ratio of 16:24 can serve as the baseline formulation. On this basis, the further addition of KH560 (4–6%) achieves higher adhesion strength experimentally. It should be noted, however, that the combined effect of the two strategies is inferred from the individually optimized results rather than directly validated by orthogonal experiments in this study; future work will confirm the synergistic effect through orthogonal experimentation. This study explored the effect of the crosslink structure and interfacial connection on the adhesion of silicone gel, providing a basis and support for the design of packaging materials for high-reliability power modules.

## 4. Materials and Methods

### 4.1. Experimental Materials

The raw materials used in this study are as follows. Self-made vinyl silicone oil (methyl terminated vinyl polysiloxane), terminal-hydrogen silicone oil (active hydrogen content 0.07 wt%, chain extender, Shandong Dayi Chemical Co., Ltd., Yantai, China), side-hydrogen-containing silicone oil (active hydrogen content 0.5 wt%, crosslinker, Shandong Dayi Chemical Co., Ltd.), platinum catalyst (2000 ppm, Dongguan Aokai new materials Co., Ltd., Dongguan, China), 1-ethynyl-1-cyclohexanol (inhibitor, Shanghai McLean Biochemical Technology Co., Ltd., Shanghai, China), silane coupling agent KH560 (γ-glycidyloxypropyl trimethoxysilane, Shanghai yuanyesheng Technology Co., Ltd., Shanghai, China), KH570 (γ-methacryloyloxysilane propyl trimethoxysilane, Shanghai Aladdin Biotechnology Co., Ltd., Shanghai, China) and A171 (vinyl trimethoxysilane, Shanghai Yuanye Biotechnology Co., Ltd., Shanghai, China).

### 4.2. Preparation Process of Silicone Gel

**Figure 12:**
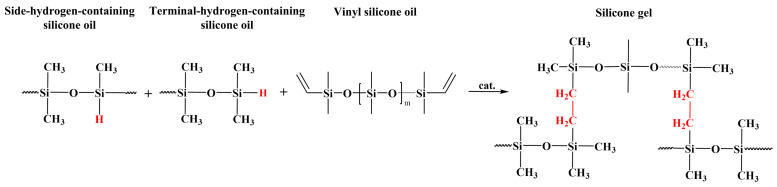
Principle of synthesis process of silicone gel.

The preparation of silicone gel is mainly realized by the hydrosilylation reaction of methyl vinyl silicone oil and hydrogen-containing silicone oil. The vinyl silicone oil is mixed and stirred with the inhibitor. A quantity of 0.5 wt% hydrogen-containing silicone oil and 0.07 wt% hydrogen-containing silicone oil are then added, stirring continued, and then platinum catalyst added, mixing evenly, and then poured into the mold for vacuum pumping. The target silicone gel material is finally obtained by curing at 110 °C for 2 h. The molar ratio of vinyl to hydrogen is kept at 1:1 during the reaction. The catalyst loading is kept strictly constant across all sample series. The reaction process is shown in Figure 12.

### 4.3. Test Method

#### 4.3.1. Adhesion Performance Test Platform

**Figure 13:**
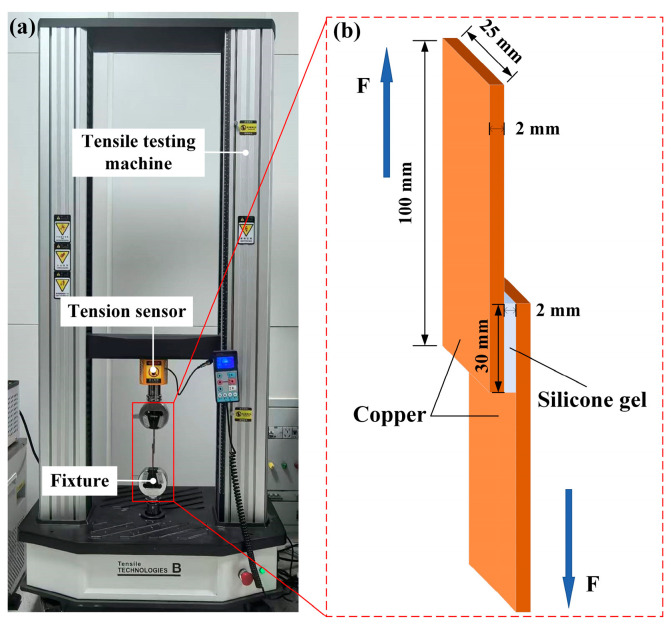
Schematic diagram of adhesion test platform. (**a**) Tensile testing machine; (**b**) Adhesion test mold.

Referring to the national standard GB/T 13936-2014 [27], a lap shear tensile test is applied to test the adhesion strength on the universal tensile testing machine, as shown in Figure 13a. Copper is selected as the representative substrate. Before the test, the copper plate is cleaned with distilled water and acetone in turn, dried at room temperature for 30 min, and then the surface polished to simulate the actual process cleanliness. The surface roughness (R_a_) of the polished copper plate is ensured to be 0.6 ± 0.05 μm to eliminate the influence of surface roughness on the test results. To minimize surface oxidation, all pretreatment and sample assembly procedures are conducted under dry ambient conditions (relative humidity < 30%), and the curing process is carried out in a vacuum oven with a vacuum level of −0.1 MPa. In order to simulate the shear stress [9] in the packaging structure, the bonding sample is prepared, injecting silicone gel between the two copper plates to form an adhesive layer with a size of 30 × 25 × 2 mm, as shown in Figure 13b. After the specimen is cured completely at 110 °C, a tensile shear test is carried out. The stretching speed of the tensile testing machine is 50 mm/min. Eight sets of overlapping samples are prepared for each sample and tested separately. The average of the eight test results is taken as the adhesion strength of the sample.

#### 4.3.2. Adhesion Failure Type Analysis

**Figure 14:**
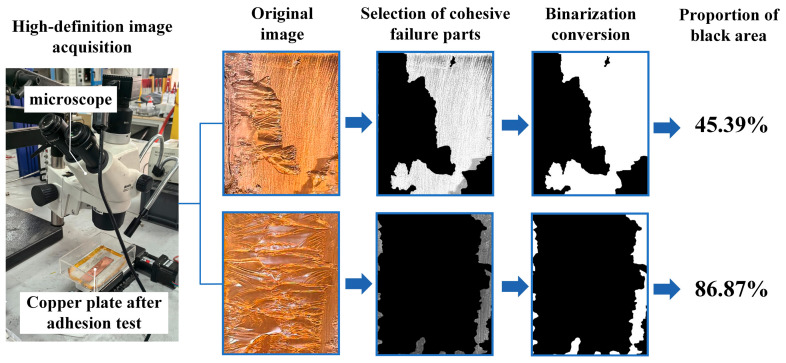
Treatment of proportion of cohesive failure area.

After the tensile shear test, the failure interface is analyzed. According to the location of failure, it can be divided into two types: interfacial failure and cohesive failure. Interfacial failure is indicated where the silicone gel is completely peeled off from the surface of the copper plate, and the interface is clear without residue. Cohesive failure reflects failure occurring inside the silicone gel, with the result that there is an obvious silicone gel residue on the surface of the copper plate. High-definition images are collected through the microscope, and the proportion of cohesive failure area is quantified and counted utilizing image processing software, so as to provide a quantitative description of the failure mode, as shown in Figure 14. Dominant interfacial failure indicates that the interface bonding strength is insufficient, while dominant cohesive failure means that the interface bonding strength has exceeded the bulk strength. A failure type analysis is conducted for the upward pulled copper plate after the tensile test. Eight failure areas are collected for each sample, and the average is taken as the failure area for that sample.

#### 4.3.3. Crosslink Density and Contact Angle Test Platform

The crosslink density is an important parameter to characterize the crosslink network structure and cohesive strength of silicone gel. The crosslink density is measured using a low-field nuclear magnetic resonance (LF-NMR) instrument (Niumag M3). This technique quantifies the crosslink density by measuring the transverse relaxation time (T_2_) of proton spins. The T_2_ value reflects the degree of constraint on molecular chain segment mobility imposed by crosslink junctions, with a shorter T_2_ corresponding to a higher crosslink density [28,29]. Silicone gel samples are cut into cylinders of approximately 10 mm in diameter and 5 mm in thickness and are then placed in NMR tubes for testing. Each sample is tested five times, and the final crosslink density is taken as the average value. The contact angle is an important parameter to characterize the surface wettability of silicone gel [30]. Contact angle measurements are performed using a visual optical contact angle meter (Kruss DSA100S, KRUSS GmbH, Hamburg, Germany). The silicone gel sample is placed flat on the sample stage, and approximately 2 μL of deionized water is dropped onto the sample surface. The droplet profile is recorded in real time by a high-speed camera, and the static contact angle is calculated. Five different positions on each sample are tested, and the final result is taken as the average.

#### 4.3.4. Hardness and Shear Modulus Test Platform

In order to evaluate the mechanical properties of silicone gel, the hardness and shear modulus of silicone gel are tested. Hardness is measured using a texture analyzer (Rapid TA, Shanghai Tengba Instrument Technology Co., Ltd., Shanghai, China). An A-type probe is pressed into the sample surface at a speed of 1 mm/s to a preset deformation depth, held for 3 s, and the maximum invasive force (gf) required to reach that depth is recorded. In this fixed-depth indentation test, a higher invasive force indicates greater resistance to deformation and thus higher hardness. Since all measurements are conducted under identical conditions, the recorded invasive force values directly and reliably reflect the relative hardness differences among samples. Eight hardness tests are conducted on the silicone gel at each ratio, and the average value taken of the results. The shear modulus is measured using the same universal tensile testing machine and identical lap shear specimens as those used for adhesion strength testing, as shown in Figure 13. The silicone gel between the two copper plates forms an adhesive layer with dimensions of 30 × 25 × 2 mm. During the test, the shear force is applied parallel to the 30 mm × 25 mm area at a constant loading rate of 50 mm/min. The stress–strain curve is recorded in real time throughout the entire loading process via the machine’s built-in “stress–strain” acquisition module. After the test, the initial approximately linear segment of the curve within the 1–5 mm displacement range is uniformly selected for shear modulus calculation. The software adopts a secant modulus algorithm, and upon inputting the specimen dimensions, it automatically outputs the shear modulus value.

#### 4.3.5. Breakdown Test Platform

**Figure 15:**
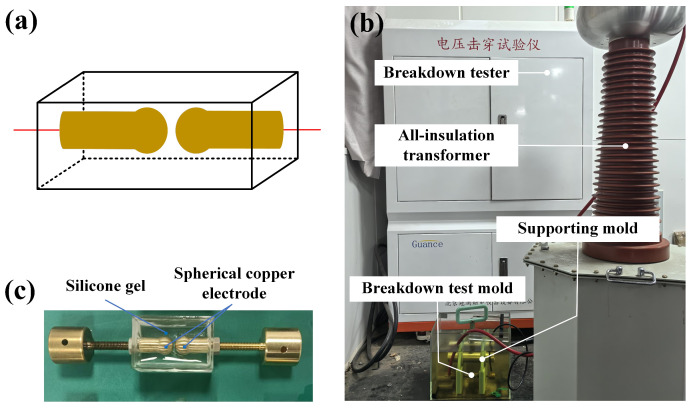
(**a**) Schematic diagram of breakdown mold; (**b**) Breakdown test platform figure; (**c**) Breakdown test mold figure.

In order to evaluate the feasibility for high-voltage power modules, an AC breakdown test platform is built to obtain the intrinsic insulation strength of silicone gel. The main parts of the platform include a 0~50 kV automatic voltage regulator, protection resistance, measurement and control system and a safety protection device, as shown in Figure 15b. A 50 Hz AC voltage is used for the test, and the voltage is boosted at a constant rate of 1 kV/s until the sample breakdown occurs. A symmetrical spherical copper electrode structure with a diameter of 22 mm as shown in Figure 15a,c is used in the test, and the electrode spacing is 1 mm. The silicone gel is injected into the cuboid uncovered glass box of the mold (the internal size is 46 × 26 × 28 mm). During the test, the breakdown test mold is supported by the support mold and immersed into the insulating oil for the test.

### 4.4. Experimental Design

#### 4.4.1. Adhesion Performance Test and Crosslink Density Test with Different Crosslink Ratios

To regulate the crosslink structure of the silicone gel, we adjust the molar ratio of active hydrogen (Si–H) provided by side-hydrogen-containing silicone oil and terminal-hydrogen-containing silicone oil. The total molar amount of Si–H from both oils is normalized to 40 parts, and the ratio of side-H to terminal-H (e.g., 16:24) directly represents their molar ratio. Under the condition of keeping the overall Vi: H molar ratio at 1:1, we prepare silicone gel with seven different side-H:terminal-H molar ratios: 10:30, 12:28, 14:26, 16:24, 18:22, 20:20 and 22:18. The corresponding masses of each hydrogen-containing silicone oil are calculated based on their active hydrogen contents (0.5 wt% for side-H and 0.07 wt% for terminal-H). Eight groups of adhesion performance tests are conducted at each ratio, and the obtained adhesion strength and adhesion failure area data are averaged. Five crosslink density tests are carried out for the silicone gel in each proportion, and the results are averaged. Eight hardness and shear modulus tests are also carried out for each proportion of silicone gel, and the results are averaged.

#### 4.4.2. Breakdown Performance Test of Silicone Gel with Different Crosslink Ratios

The representative crosslink silicone gel is selected for breakdown strength testing to observe whether changing the crosslink ratio will have a significant impact on the breakdown field strength.

#### 4.4.3. Adhesion Performance Test and Crosslink Density Test with Different Silane Coupling Agents

Three kinds of silane coupling agents, KH560, KH570 and A171, are selected for the test. Each silane coupling agent is added to the silicone gel system in four proportions of 2%, 4%, 6% and 8% of the mass of vinyl silicone oil. A silane coupling agent is added by integral blend. The order of addition is after adding two types of hydrogen-containing silicone oil and before adding the catalyst. Eight groups of adhesion performance tests are carried out at each ratio of each coupling agent, and the obtained adhesion strength and adhesion failure area data are averaged. Five crosslink density tests are carried out for the silicone gel in each proportion, and the results are averaged.

#### 4.4.4. Contact Angle Test of Silicone Gel with Different Silane Coupling Agents

Based on surface wetting theory, the contact angle of silicone gel with different amounts of silane coupling agents is tested to evaluate the effect of coupling agents on the surface wettability, and to determine the mechanism of improving the adhesion performance of silicone gel.

#### 4.4.5. Breakdown Performance Test of Silicone Gel with Different Silane Coupling Agents

Because the silane coupling agent is an exogenous molecule, its addition may adversely affect the intrinsic electrical insulation properties of silicone gel. Therefore, it is necessary to evaluate the insulation performance of the silicone gel system with the silane coupling agent by breakdown testing. Eight independent samples are prepared for breakdown testing of each sample, and Weibull distribution analysis is carried out for the eight breakdown field strength values [31,32]. The breakdown field strength at 63.2% is taken as the breakdown field strength of the sample.

## Data Availability

The original findings presented in this work are fully included in the article. Further details may be obtained by contacting the corresponding authors.
